# The distribution of neuroligin4, an autism-related postsynaptic molecule, in the human brain

**DOI:** 10.1186/s13041-023-00999-y

**Published:** 2023-02-06

**Authors:** Akie Toya, Masahide Fukada, Eiko Aoki, Tohru Matsuki, Masashi Ueda, Shima Eda, Yoshio Hashizume, Akio Iio, Shigeo Masaki, Atsuo Nakayama

**Affiliations:** 1 Department of Cellular Pathology, Institute for Developmental Research, Aichi Developmental Disability Center, Kasugai, 480-0392 Japan; 2 Department of Neurochemistry, Nagoya University Graduate School of Medicine, Nagoya, 466-8560 Japan; 3 Institute for Medical Science of Aging, Aichi Medical University, Nagakute, 480-1195 Japan

**Keywords:** ASD, NLGN4, Immunohistochemistry, Periventricular nucleus, Supraoptic nucleus, Arginine-vasopressin, Oxytocin

## Abstract

*NLGN4X* was identified as a single causative gene of rare familial nonsyndromic autism for the first time. It encodes the postsynaptic membrane protein Neuroligin4 (NLGN4), the functions and roles of which, however, are not fully understood due to the lack of a closely homologous gene in rodents. It has been confirmed only recently that human NLGN4 is abundantly expressed in the cerebral cortex and is localized mainly to excitatory synapses. However, the detailed histological distribution of NLGN4, which may have important implications regarding the relationships between NLGN4 and autistic phenotypes, has not been clarified. In this study, we raised specific monoclonal and polyclonal antibodies against NLGN4 and examined the distribution of NLGN4 in developing and developed human brains by immunohistochemistry. We found that, in the brain, NLGN4 is expressed almost exclusively in neurons, in which it has a widespread cytoplasmic pattern of distribution. Among various types of neurons with NLGN4 expression, we identified consistently high expression of NLGN4 in hypothalamic oxytocin (OXT)/vasopressin (AVP)-producing cells. Quantitative analyses revealed that the majority of OXT/AVP-producing neurons expressed NLGN4. NLGN4 signals in other large neurons, such as pyramidal cells in the cerebral cortex and hippocampus as well as neurons in the locus coeruleus and the raphe nucleus, were also remarkable, clearly contrasting with no or scarce signals in Purkinje cells. These data suggest that NLGN4 functions in systems involved in intellectual abilities, social abilities, and sleep and wakefulness, impairments of which are commonly seen in autism.

**Supplementary Information:**

The online version contains supplementary material available at 10.1186/s13041-023-00999-y.

## Introduction

Autistic spectrum disorders (ASDs) are pervasive behavioral disorders characterized by impaired communication and social activities as well as repetitive and obsessive behaviors. Intensive studies in clinical genetics have revealed that genetic changes contributing to ASD pathogenesis are highly heterogeneous, and the number of genes disrupted de novo, hence responsible for sporadic ASDs with a severe phenotype, is approximately 400 [1]. Among hundreds of genes considered to be involved in ASD pathogenesis, the *NLGN4X* gene has been identified for the first time as a single causative gene for nonsyndromic, familial ASDs, along with the related *NLGN3* gene [2]. To date, several *NLGN4X* variants associated with ASD and/or intellectual disability (ID) mainly in an X-linked recessive inheritance pattern have been reported, yet *NLGN4X* variants seem responsible for the pathogenesis in a limited population of ASD/ID [3–10].

*NLGN4* is one of the highly conserved *NLGN* genes that encode neuroligin (NLGN) 1–5, all of which are regarded as postsynaptic cell-adhesion molecules. Knowledge about the functions of NLGNs has been derived mainly from studies on murine NLGNs, and they are considered essential for synaptic functions but not for primary synapse formation through interactions with presynaptic neurexins (NRXNs) [11, 12]. Although the specific functions of each NLGN are largely unknown, NLGN2 is considered to be involved mainly in inhibitory synaptic functions [13]. NLGN1, NLGN3, and NLGN4, which are structurally more similar to each other than to NLGN2, are involved mainly, even if not exclusively, in excitatory synaptic functions [11]. NLGN5, which is almost identical to NLGN4 and is encoded on the Y chromosome, is now designated NLGN4Y, and knowledge about its function is even more limited [14, 15]. The mouse homolog of *NLGN4*, which was initially not found in mouse genome assemblies, has been shown to be divergent from *NLGN4* in other species and even between different mouse strains [10, 16, 17]. Although *Nlgn4* KO mice exhibit a selective perturbation of social behavior and vocalization and are reported as genetic mouse models of ASD [18], accumulating lines of evidence have strongly suggested that human and mouse NLGN4 do not share sufficient structural homology and function for *Nlgn4* KO mice to be a viable ASD model [10, 17].

The lack of a closely homologous gene in rodents has hampered research on NLGN4, and it has only recently been reported by Marro et al. that NLGN4 was detected at the highest level in the cerebral cortex [17]. Unfortunately, however, detailed information about the distribution at the histological level was not available in the study, since the antibodies did not work for fixed tissue. In this study, we generated two kinds of antibodies against NLGN4 that are applicable for immunohistochemistry and examined NLGN4 distribution in developing and developed human brains using them. We revealed that NLGN4 is expressed almost exclusively in neurons in the brain and is especially enriched in several anatomical regions, including hypothalamic nuclei, and a large fraction of NLGN4 is located in the cytoplasm, despite its identity as a postsynaptic membrane protein. These results provide new insights into potential roles of NLGN4 in hypothalamic nuclei involved in social functions and in additional extrasynaptic functions.

## Methods

### Antibodies

We generated a rabbit polyclonal antibody against human NLGN4 (anti-NL4-p1) in a standard immunization protocol. Briefly, we immunized a rabbit (Chubu-Kagaku Co., Nagoya, Japan) with a synthetic peptide corresponding to amino acids 647–660 (PKHSKDPHKTGPED) of NLGN4 (NP-851849). We also generated a mouse monoclonal antibody against human Neuoroligin-4, named anti-NL4-m1, by immunizing three 8-week-old balb/c females with keyhole limpet hemocyanin–conjugated synthetic peptides corresponding to amino acids 111–122 (PQHLDERSLLHD). B cells were collected from lymph nodes and fused with SP2/0-AG14 myeloma cells with polyethylene glycol 4000. Clone selection was performed by ELISA and Western blot analyses. Both anti-NL4-p1 and anti-NL4-m1 antibodies purified by protein G affinity chromatography were used in the following experiments. The commercially available antibodies used in this study were as follows: AB911 against oxytocin (Chemicon International, Temecula, CA), AB1565 against vasopressin (Chemicon), A8592 against FLAG (Sigma‒Aldrich, St. Louis, MO), CLT9002 against α-tubulin (Cedarlane, Burlington, Ontario, Canada), AC-15 against β-actin (Abcam, Cambridge, MA, USA), GTX116674 against Neuroligin4 (GeneTex International Corporation, Hsinchu, Taiwan), F1804 against FLAG (Sigma‒Aldrich), Alexa Fluor 488–conjugated goat anti-mouse IgG (Molecular Probes, Carlsbad, CA, USA), and Alexa Fluor 568–conjugated goat anti-rabbit IgG (Molecular Probes).

### Construction of expression vectors, transfection and Western blotting

The cDNA fragments encoding human NLGN1 (amino acid residues 46-823; GenBank Accession No. NM_014932), NLGN2 (amino acid residues 16-835; GenBank Accession No. AF376802), NLGN3 (amino acid residues 35-828; GenBank Accession No. BC051715), and NLGN4 (amino acid residues 39-816; GenBank Accession No. AF376803) were cloned into multicloning sites of the p3xFLAG-CMV-9 vector (Sigma) to yield p3xFLAG-NLs.

HEK293 cells were cultured in DMEM (Nacalai Tesque, Kyoto, Japan) with 10% fetal bovine serum (Gibco, Grand Island, NY, USA) and 1% penicillin and streptomycin (Wako, Osaka, Japan). Transient transfection of expression vectors p3XFLAG-NLs into HEK293 cells was carried out using polyethylenimine MAX (Polysciences, Warrington, PA, USA) according to the manufacturer’s directions. Western blot analyses were performed as described previously [19].

### Human induced pluripotent stem cell (iPSC) culture and generation of NLGN4X knockout iPSC clones

The human iPSC Line 610B1 was obtained from RIKEN BioResource Center (Tsukuba, Japan). The iPSCs were maintained in a pluripotent undifferentiated state on mitomycin C-treated mouse SNL feeder cells in ES medium [DMEM/F12 with 20% StemSure Serum Replacement, 0.5 mM StemSure monothioglycerol, 0.8% MEM nonessential amino acids solution, 2 mM l-alanyl-l-glutamine, 0.5% penicillin and streptomycin, and 10 ng/mL fibroblast growth factor (FGF) (FUJIFILM Wako Pure Chemical Co., Tokyo, Japan)]. To generate *NLGN4X* knockout (KO) iPSC clones, we applied CRISPR/Cas9-mediated genome editing with dual RNP complexes prepared by incubation with each CRISPR RNA (crRNA), trans-activating CRISPR RNA (tracrRNA), and Alt-R^®^ CRISPR‒Cas9 protein (IDT, Coralville, IA) with reference to previous reports [20, 21]. Two gRNAs, gRNA1 (CAGACCGGGGTGAACAACAA) complementary to exon 2 and gRNA2 (TGATTCCAAACACACTGACG) complementary to exon 6 of *NLGN4X,* were designed using Benchling (https://www.benchling.com) (Additional file 1: Fig. S1B). To assess *NLGN4X* knockout in iPSCs, genomic PCR was performed with the following primer sets: pEx2f (CTCGCCTCTGGGCTTTGTCTCCTTGGAGCC), pEx2r (TCATCAAAGTATCCAAATTGGCG), pEx6f (TTAAGTGTCACCATTGCCGTC), and pEx6r (GGAAAACACCAACGATAAGGG) (Additional file 1: Fig. S1B).

### Human tissue samples

All human brain samples used for immunohistochemical studies were obtained by autopsy at the Central Hospital of Aichi Developmental Disability Center, Nagoya First Red Cross Hospital, and Nagoya Second Red Cross Hospital. Cases whose clinical records indicated diseases involving the central nervous system were excluded, and all brain specimens from the remaining cases were re-examined by pathologists (YH and AN) to confirm that they had no obvious brain damage or abnormalities. After exclusion, ten neonatal, two infantile, and eight adult brains remained as subjects for immunohistochemical studies. A summary of the cases is shown in Table 1A, and information on each case is listed in Table 1B.

**Table 1:** Summary and list of cases

(A) Summary of cases		
Age	Numbers of cases	
	Male	Female
Preterm neonate (GA 30–35 weeks)	1	1
Term neonate (PA 0–30 days)	6	2
Infant (4–9 months)	1	1
Adult (17–45 years)	5	3

(B) List of cases				
ID	Age^a^	Sex	Diagnoses	Figure(s)^b^
N1	30 w (0 d)	♂	Cystic hygroma	4G, Additional file 2: Fig. S2A–D
N2	33 w (0 d)	♀	Premature baby, neonatal asphyxia	3F
N3	37 w (5 d)	♂	Diaphragmatic hernia	6F
N4	38 w (4 w)	♂	Diaphragmatic hernia	5E
N5	38 w (0 d)	♂	Potter syndrome	3E, 5C, F
N6	39 w (1 d)	♀	Sick baby born to a mother with gestational diabetes	6B
N7	39 w (3 d)	♂	SIDS	4C
N8	40 w (0 d)	♂	Neonatal aspiration syndrome	2F, G
N9	40 w (1 d)	♂	Pulmonary hypoplasia	6G
N10	43 w (4 w)	♀	SIDS	2H–J, 4H, 5A, B, D, 6H
I1	48 w (16 w)	♀	Fetal pleural effusion	3G, H, 4D
I2	76 w (36 w)	♂	Allergy, asphyxia	
A1	17 y	♂	Acute heart failure	6A
A2	28 y	♀	Shock after delivery	4A, 7A
A3	30 y	♂	Dermatomyositis	2A, B
A4	31 y	♀	Anorexia	3D, 4B, E, 6D, E, Additional file 2: Fig. S2G, H
A5	32 y	♀	Unspecified sudden death	2E
A6	40 y	♂	DOA	2C, D, 3A–C, 4F
A7	45 y	♂	DOA	6C, Additional file 2: Fig. S2EF
A8	45 y	♂	Rupture of the esophageal varices	

*GA* gestational age, *PA* postnatal age

^a^The gestational age, with the postnatal age written in parenthesis for cases N1-10, I1 and I2

^b^The numbers correspond to the numbered figures included in this article

Stored formalin-fixed and paraffin-embedded brain tissues were sectioned at 6 μm thickness, and the sections were used for immunohistochemistry and hematoxylin and eosin staining. Research use of autopsy samples was permitted with informed consent from the next of kin, and the study was carried out with the authorization of the Ethics Committee of Institute for Developmental Research, Aichi Developmental Disability Center, endorsed as #11-06.

### Immunohistochemistry and immunocytochemistry

The staining procedure was performed as described previously [22]. Briefly, antigen retrieval was performed by heat treatment in 10 mM citrate buffer (pH 6.0) in a microwave oven for 5 min (two cycles) at 600 W. After blocking with 4% normal goat serum in PBS/0.1% Triton-X, brain sections on glass slides were incubated with primary antibodies at 4 °C overnight. The dilutions of primary antibodies were as follows: 500 × (anti-NL4-p1), 5000x (anti-oxytocin), 5000x (anti-vasopressin), and 1000 × (anti-NL4-m1). For visualization of the antigens, a VECTASTAIN Elite ABC-HRP Kit (Vector Labs., Burlingame, CA, USA) was applied following the manufacturer’s protocol. In fluorescent immunocytochemistry, we applied Alexa Fluor 488–conjugated goat anti-mouse IgG at a 500 × dilution and Alexa Fluor 568–conjugated goat anti-rabbit IgG at a 500 × dilution. Fluorescent images were obtained by a confocal laser scanning microscope LSM880 (Carl Zeiss, Göttingen, Germany).

## Results

### Generation and characterization of NLGN4-specific antibodies

**Fig. 1:**
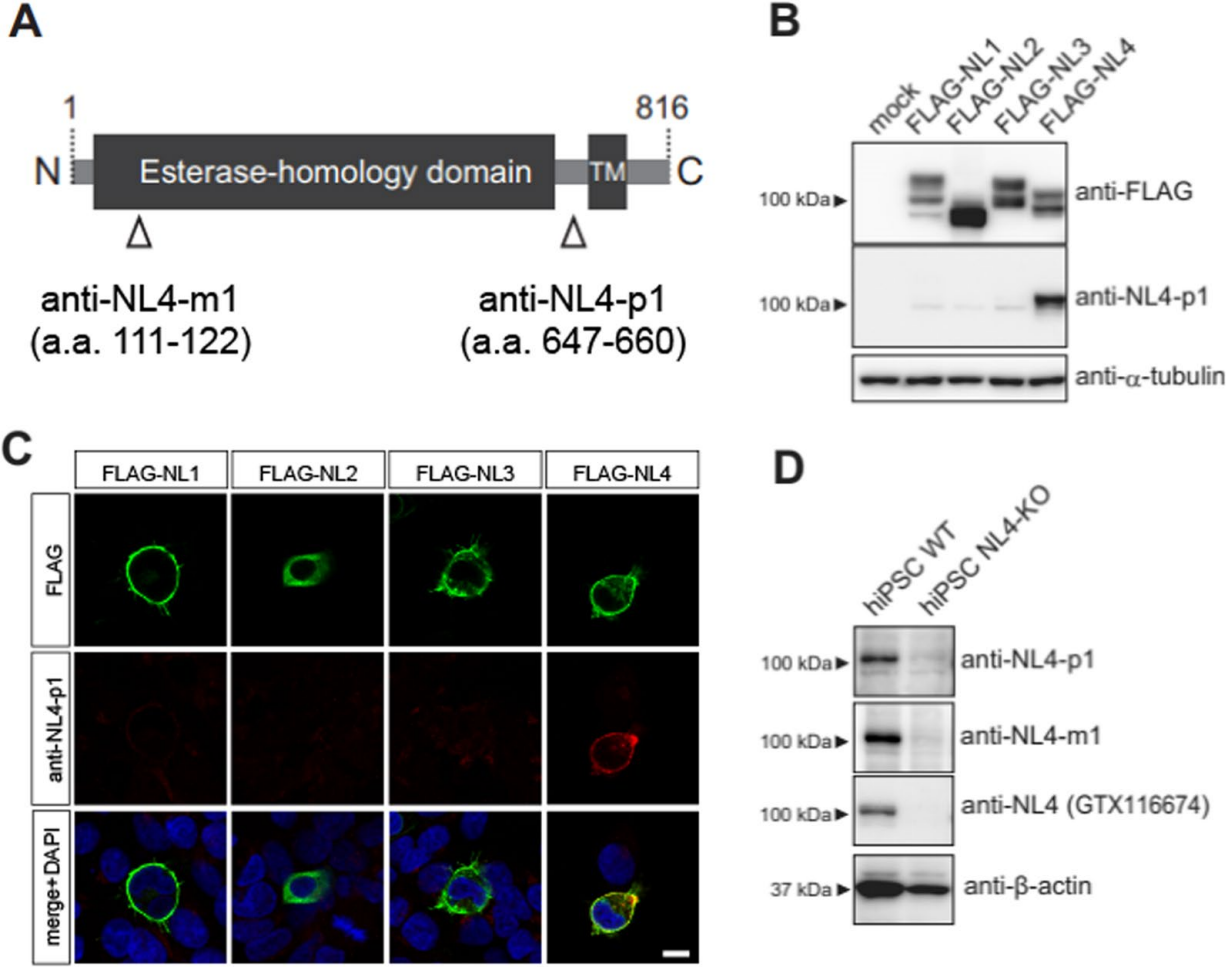
Production and characterization of antibodies against human NLGN4. **A** The domain structure of human NLGN4. The positions of immunogens used to produce anti-NLGN4 antibodies are shown at the bottom. **B** Anti-NL4-p1 antibodies specifically react with NLGN4 but not with the other NLGNs in Western blotting. Lysates of HEK293 cells overexpressing FLAG-tagged NLGN1 (FLAG-NL1), NLGN2 (FLAG-NL2), NLGN3 (FLAG-NL3), or NLGN4 (FLAG-NL4) were electrophoresed and immunoblotted with anti-FLAG and anti-NL4-p1 antibodies. Anti-α-tubulin antibodies were used as a loading control. Anti-NL4-p1 antibodies reacted with FLAG-NL4 and did not cross-react with FLAG-NL1, 2, and 3. Lysates of HEK293 cells transfected with empty FLAG vector or pcDNA3.1 were used as a negative control (mock). **C** Anti-NL4-p1 antibodies specifically react with NLGN4 but not with other NLGNs in fluorescent immunocytochemistry. HEK293 cells expressing FLAG-NL1, 2, 3, or 4 were immunolabeled with anti-FLAG (green) and anti-NL4-p1 (red) antibodies. Merged images with DAPI nuclear staining (blue) are shown at the bottom. Anti-FLAG antibodies recognized FLAG-NL1, 2, 3, and 4 expressed on the cell membrane. Anti-NL4-p1 antibodies recognized only FLAG-NL4 but not FLAG-NL1, 2, and 3. Scale bar = 10 μm. **D** Anti-NL4-p1 and anti-NL4-m1 antibodies react with endogenous NLGN4 expressed in human iPSCs. Cell lysates from human iPSC clone 610B1 (hiPSC WT) and its *NLGN4X* knockout derivative (hiPSC NL4-KO) were electrophoresed and immunoblotted with anti-NL4-p1, anti-NL4-m1, anti-NLGN4 (GTX 116674), and anti-β-actin antibodies. Endogenous NLGN4 proteins in original hiPSC WT lysates were detected with anti-NL4-p1, anti-NL4-m1, and GTX 116674 antibodies, whereas no signals were detected in hiPSC NL4-KO lysates

We established NLGN4-specific polyclonal and monoclonal antibodies, named anti-NL4-p1 and anti-NL4-m1, respectively, targeting unique epitopes on NLGN4 to avoid cross reactions with the other NLGNs (Fig. 1A). As expected, both antibodies reacted with full-length human NLGN4 in Western blotting analyses but not with full-length human NLGN1, 2, or 3 when they were overexpressed in HEK293 cells (Fig. 1B; Additional file 1: Fig. S1A). We further confirmed the specificity in immune-fluorescent cytochemistry (Fig. 1C). Then, we assessed the sensitivities of the antibodies against endogenous NLGN4 by Western blotting. We, however, could not detect NLGN4 bands with anti-NL4-p1 or anti-NL4-m1 in several different human brain samples. We reasoned that sensitivities of the antibodies were not enough high to detect a low amount of NLGN4 in brain tissue lysates due to the low abundance of NLGN4-expressing cells. Therefore, we analyzed cell lysates prepared from human iPSCs, which are known to express NLGN4 [23]. Both anti-NL4-p1 and anti-NL4-m1 visualized bands just above 100 kDa in accordance with the molecular size of NLGN4 reported previously [17], similar to GTX116674, a commercially available anti-NLGN4 antibody (Fig. 1D). These bands were not observed in lysates prepared from *NLGN4X* KO iPSCs (Fig. 1D; Additional file 1: Fig. S1B, C), confirming that the bands recognized by anti-NL4-p1 and anti-NL4-m1 were derived from endogenous NLGN4.

### Distribution of NLGN4 in the brain

In initial studies, we confirmed that anti-NL4-p1 and anti-NL4-m1 provided very similar staining patterns with occasionally clearer images by anti-NL4-p1, and the signals were observed exclusively in neuronal lineage cells. In addition, both antibodies labeled the whole neuronal cell bodies but did not react in a cell-membrane-specific manner, yet NLGNs are regarded as postsynaptic membrane proteins. The pattern was, however, consistent with previous reports revealing subcellular distributions of other endogenous NLGNs in the tissue section [24–26]. We also noticed that the intensities of signals varied among subtypes of the neuron. Therefore, we will present detailed data divided into subsections, i.e., “The forebrain”, “The midbrain”, and “The hindbrain”, in that order. Representative images stained with anti-NL4-p1 at 1:500 dilution will be mainly shown unless otherwise mentioned. In addition, a summary of data in major anatomical structures is presented in Table 2.

**Table 2:** Summary of NLGN4 expression in the brain

Anatomical location^a^		Major functions	Neonates and Infants	Adults
Forebrain	Cerebral cortex Hippocampus, Pyramidal cells Hippocampus, Granule cells Amygdala Caudate Putamen Globus pallidus	Complex integral function Memory Memory Fear Motor modification Motor modification Motor modification	+ (6/8), ± (2/8) + (4/6), ± (2/6) + (3/7), ± (3/7), – (1/7) ND + (5/8), – (3/8) – (7/8), + (1/8) + (4/8), – (4/8)	+ (6/7), ± (1/7) + (4/6), ± (1/6), – (1/6) + (3/6), ± (1/6), – (2/6) – (4/4) + (2/6), ± (2/6), – (2/6) – (5/5) – (5/5)
	Thalamus Hypothalamus	Sensory modification Mood and emotion	+ (7/7) + (8/8)	+ (4/5), ± (1/5) + (5/5)
Midbrain	Substantia nigra Red nucleus III	Motor modification Upper-limb movement Ocular movement	+ (5/9), ± (2/9), – (2/9) – (5/5) + (4/6), ± (2/6)	– (4/7), ± (1/7), + (2/7) – (7/7) + (3/7), ± (1/7), – (3/7)
Hindbrain	Purkinje cells Granule cells Dentate nucleus Raphe nucleus Locus coeruleus Inferior olive XII	Motor regulation Motor regulation Motor regulation Arousal, wakefulness Arousal, wakefulness Motor regulation Tongue movement	+ (8/12), ± (4/12) – (12/12) + (6/10), ± (3/10), – (1/10) + (8/8) + (8/9), ± (1/9) + (11/11) + (11/11)	– (4/4) – (3/4), ± (1/4) + (2/4), ± (2/4) + (6/7), – (1/7) + (6/6) + (5/6), – (1/6) + (5/6), ± (1/6)

+ :positive; ± :weakly or partially positive; –:negative; the number of cases regarded as + , ± , or – (numerator) with respect to the number of available cases (denominator) is presented in parentheses

^a^The structures with consistent NLGN4 expression are underlined

#### The forebrain

**Fig. 2:**
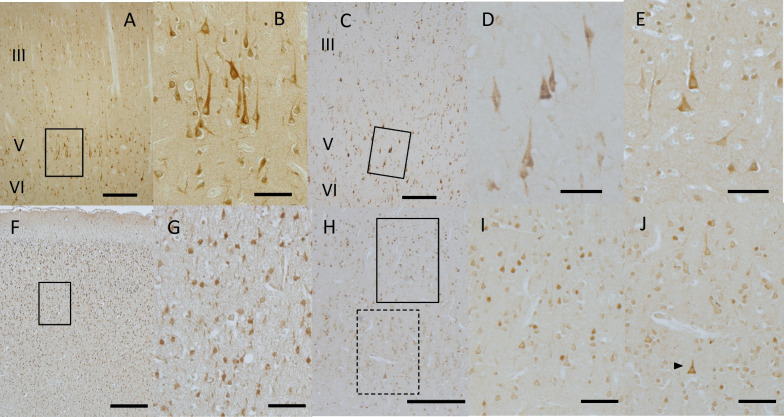
Immunohistochemical distribution of NLGN4 in the human cerebral cortex. **A** A representative image of the adult human cerebral cortex (case ID: A3). The positions of cortical layers III, V, and VI are indicated. **B** A magnified view of the boxed area in **A**. **C** A representative image of the adult human cerebral cortex (case ID: A6) examined with ant-NL4-m1. The positions of cortical layers III, V, and VI are indicated. **D** A magnified view of the boxed area in **C**. **E** A high-magnification view of layer V in the adult female cerebral cortex (case ID: A5). Remarkable NLGN4 expression was observed as in the male cerebral cortex. **F** A representative image of the neonatal cerebral cortex (case ID: N8). The cortical layer structure is unclear, and NLGN4-positive neurons appear evenly distributed. **G** A magnified view of the boxed area in **F**. Most neurons expressed NLGN4. **H** Another representative image of the neonatal cerebral cortex (case ID: N10). An incomplete layer structure was present in this case. **I** A magnified view of the solid-boxed area in **H**. Granular cells in layer II and pyramidal cells in layer III expressed NLGN4 at various levels. **J** A magnified view of the dashed-boxed area in **H**. Large pyramidal cells in layers V and VI expressed NLGN4 at various levels. Intense NLGN4 signals were noted in a pyramidal cell (black arrowhead) as in the adult cerebral cortex. Scale bars = 200 μm (**A**, **F**, **H**), 50 μm (**B**–**E**, **G**, **I**, **J**)

In the adult cerebral cortex, NLGN4 signals were detected similarly in most neurons by anti-NL4-p1 and by anti-NL4-m1 and in both male and female samples (Fig. 2A–E). In particular, intense staining signals were observed in large pyramidal neurons in layer V (Fig. 2B, D, E). Neuronal NLGN4 expression was identified in all areas of the cerebral cortex we observed, although we did not thoroughly examine the cerebral cortical areas. In neonatal brains, most neurons that do not yet constitute the clear layer structure in the cortex showed NLGN4 signals (Fig. 2F, G). In a neonatal brain with further development, an ambiguous layer structure appeared, and intense NLGN4 signals were detected in some large pyramidal cells, as in the adult brain (Fig. 2H–J).

**Fig. 3:**
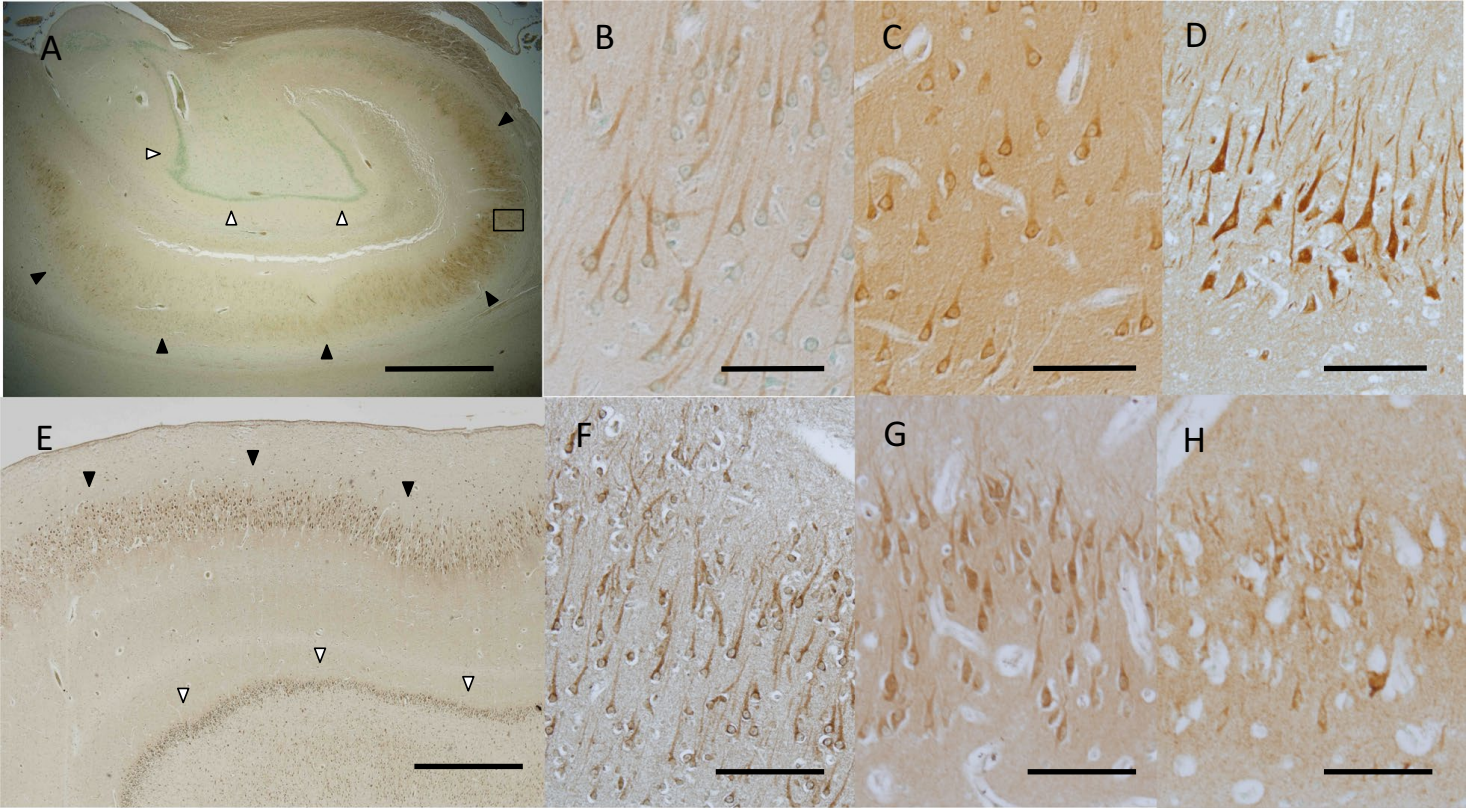
Immunohistochemical distribution of NLGN4 in the human hippocampus. **A** A representative image of the adult hippocampus (case ID: A6). Intense NLGN4 signals were observed in the pyramidal cell layer (black arrowheads), while no signals were observed in the granule cell layer (white arrowheads). **B** A magnified view of the boxed area in **A**. Clear NLGN4 signals were observed in pyramidal neurons in the hippocampus. **C** A high-magnification view of the pyramidal cell layer in case A6 examined with anti-NL4-m1 (1:1000 dilution). Clear signals with relatively high background were observed. **D** A high-magnification view of the pyramidal cell layer in another adult brain (case ID: A4). Intense signals were observed in most pyramidal neurons. **E** A representative image of the neonatal hippocampus (case ID: N5). Intense NLGN4 signals were observed in the granule cell layer (white arrowheads) as well as in the pyramidal cell layer (black arrowheads). **F** A high-magnification view of the pyramidal cell layer in another neonatal brain (case ID: N2). Intense NLGN4 signals were observed in most pyramidal neurons. **G** A high-magnification view of the pyramidal cell layer in the infantile brain (case ID: I1). **H** A high-magnification view of the pyramidal cell layer in case I1 examined with anti-NL4-m1 (1:1000 dilution). Clear NLGN4 signals were observed in pyramidal neurons, as in **G**. Scale bars = 1 mm (**A**), 100 μm (**B**–**D**, **F**–**H**), 500 μm (**E**)

In the adult hippocampus, the pyramidal cell layer was immunolabeled in all available cases, while the granule cell layer was occasionally negative for NLGN4 (Fig. 3A). Most hippocampal pyramidal cells were clearly positive for NLGN4 regardless of the type of antibody and the gender of the donor (Fig. 3B–D). In all neonatal brains, both pyramidal cells and granular cells were well stained (Fig. 3E, F). In the infantile brain, pyramidal cells were positive for NLGN4 (Fig. 3G, H), but granular cells were scarcely stained in one case (not shown).

Neurons in additional telencephalic structures, such as the amygdala, the putamen, and the globus pallidus, were not labeled in adult brains, while immature neurons or precursors in the putamen and pallidus were labeled by anti-NLGN4 antibodies in some neonatal and infantile brains (not shown). Neurons in the caudate among the deep telencephalic structures were occasionally stained by the antibodies (not shown).

**Fig. 4:**
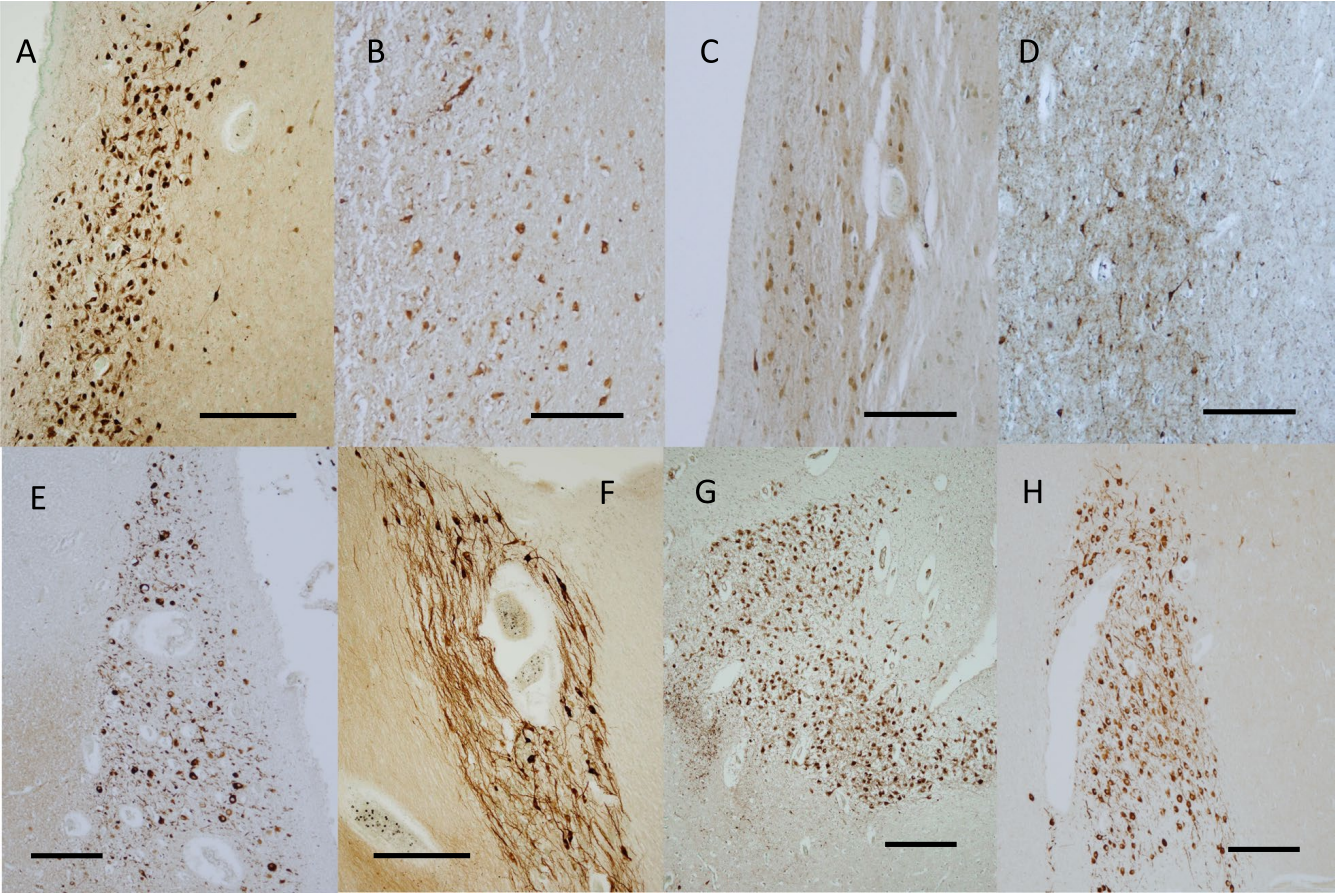
Immunohistochemical distribution of NLGN4 in the human hypothalamus. **A**, **B** Representative images of the adult paraventricular nucleus (PVN) (case ID: A2 for **A**, A4 for **B**). **C** A representative image of the neonatal PVN (case ID: N7). **D** A representative image of the infantile PVN (case ID: I1). **E**, **F** Representative images of the adult supraoptic nucleus (SON) (case ID: A4 for **E**, A6 for **F**). **G**, **H** Representative images of the neonatal SON (case ID: N1 for **G**, N10 for **H**). Intense NLGN4 signals were observed in all available PVNs and SONs. Scale bars = 200 µm (**A**–**D**, **F**), 250 µm (**E**, **G**, **H**)

In the diencephalic region, the most remarkable immunolabeled signals were observed in the paraventricular nucleus (PVN) and the supraoptic nucleus (SON) of the hypothalamus. Most, if not all, neurons were labeled by anti-NLGN4 antibodies in all available cases regardless of their developmental stages, kinds of antibodies, and sexes (Fig. 4A–H; Additional file 2: Fig. S2A–D). As shown in Fig. 4F, NLGN4 signals were remarkable in extended neurites in some cases (Fig. 4F).

**Fig. 5:**
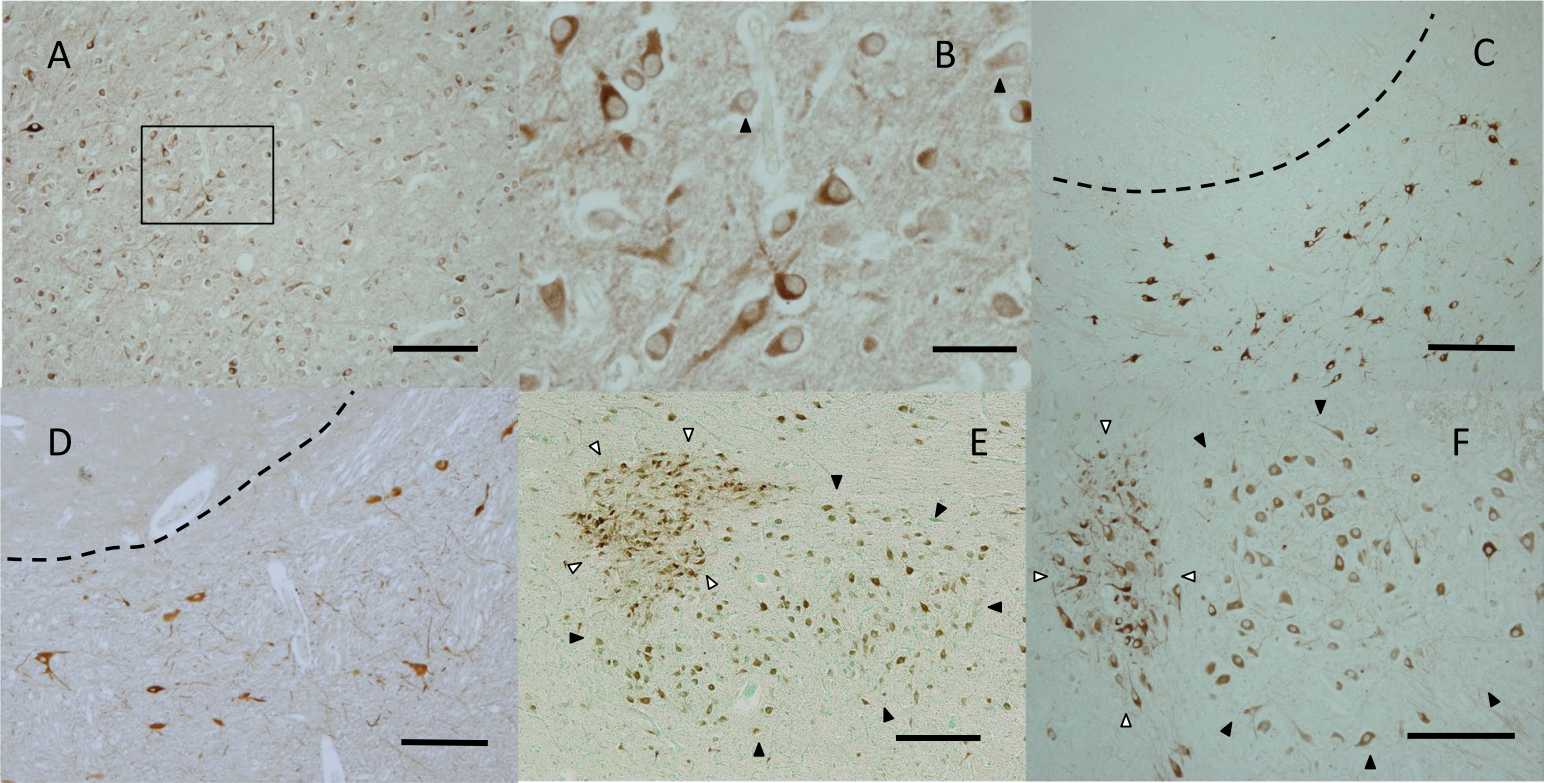
Immunohistochemical distribution of NLGN4 in the human thalamus and midbrain. **A** A representative image of the neonatal thalamus (case ID: N10). NLGN4-positive cells appeared sparsely distributed. **B** A magnified view of the boxed area in **E**. NLGN4-negative to weakly NLGN4-positive neurons (black arrowheads) were located among NLGN4-positive neurons. **C** A representative image of the neonatal red nucleus and midbrain reticular formation (case ID: N5). Intense NLGN4 signals were observed in large cells in the midbrain reticular formation but not in the red nucleus. The boundary of the red nucleus is indicated by the dashed line. **D** Another image of the neonatal red nucleus and midbrain reticular formation (case ID: N10). The boundary of the red nucleus is indicated by the dashed line. **E**, **F** Representative images of the neonatal oculomotor nucleus (case ID: N4 for **E**, N5 for **F**). In both cases, intense NLGN4 signals were observed in the accessory nucleus (white arrowheads), and moderate to intense NLGN4 signals were observed in the main nucleus (black arrowheads). Scale bars = 100 µm (**A**, **F**), 25 µm (**B**), 200 µm (**C**, **D**, **E**)

Neurons in the thalamus of almost all cases were stained by the antibodies, yet NLGN4-positive neurons were sparsely distributed (Fig. 5A). At higher magnification, considerable numbers of neurons with very weak signals were identified (Fig. 5B).

#### The midbrain

Large neurons in the medial reticular formation were labeled intensely by anti-NLGN4 antibodies, which gave a sharp contrast with those lacking the signals in the red nucleus (Fig. 5C, D). Neurons in the oculomotor nucleus were labeled in all neonatal and infantile cases and approximately half of adult cases (Fig. 5E, F, Additional file 2: Fig. S2E, F). We noticed that signals in the accessory nucleus (Edinger-Westphal) looked more remarkable than those in the main nucleus (Fig. 5E, F). Neurons in the substantia nigra were weakly labeled in neonates and infants and in some adult cases (not shown).

#### The hindbrain

**Fig. 6:**
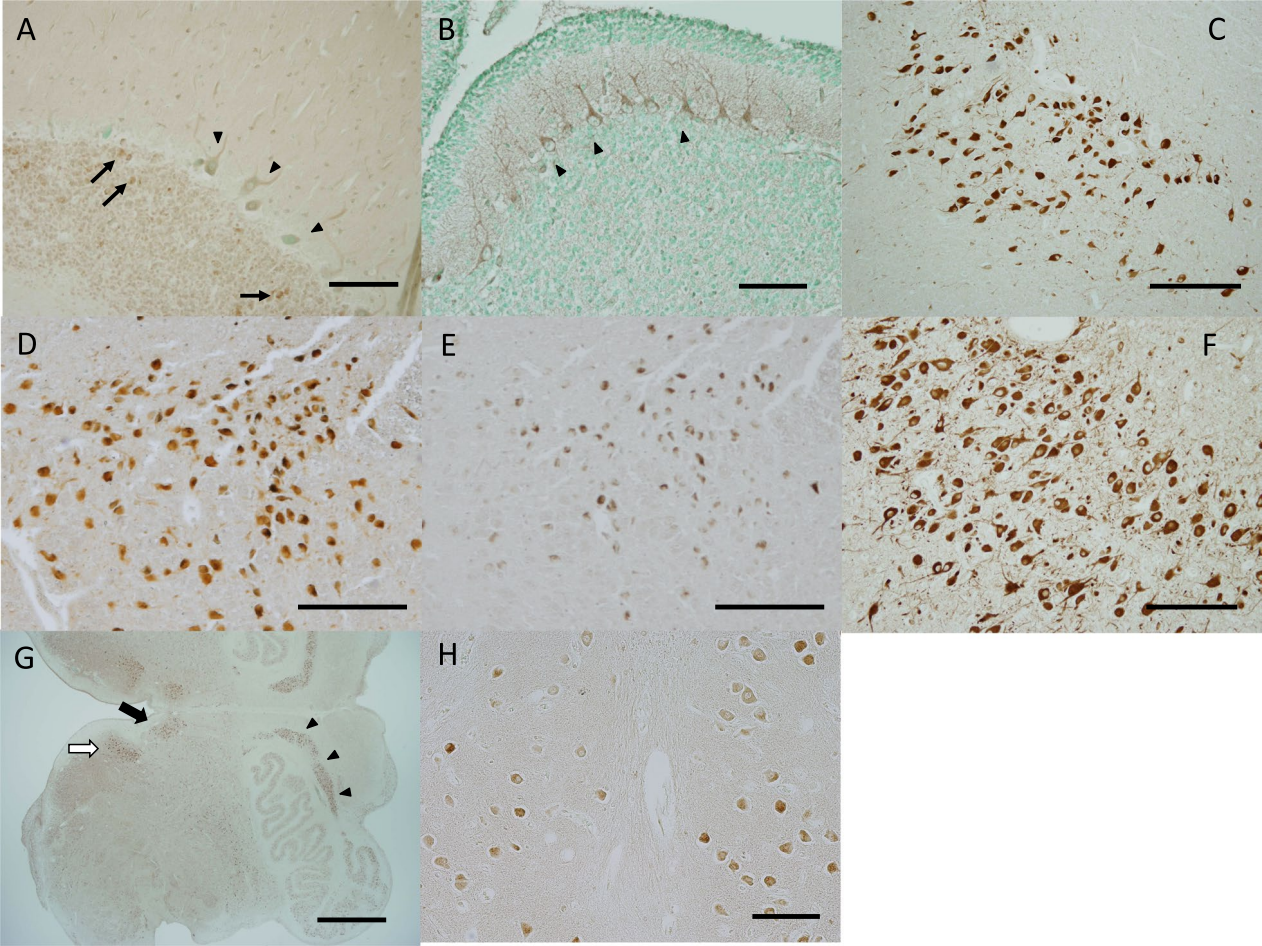
Immunohistochemical distribution of NLGN4 in the human cerebellar cortex, pons and medulla oblongata. **A** A representative image of the adult cerebellar cortex (case ID: A1). Very weak NLGN4 signals were observed in Purkinje cells (black arrowheads), and moderate NLGN4 signals were observed in some putative Golgi cells in the granule cell layer (black arrows). **B** A representative image of the neonatal cerebellar cortex (case ID: N6). In contrast to adult cases, moderate NLGN4 signals were observed in Purkinje cells (black arrowheads). **C**, **D** Representative images of the adult locus coeruleus (case ID: A7 for **C**, A4 for **D**). **E** A negative control image of a section adjacent to that presented in **D** as a reference for endogenous pigmentation caused by neuromelanins. **F** A representative image of the neonatal locus coeruleus (case ID: N3). In both adult and neonatal cases, intense NLGN4 signals were observed in most neurons of the locus coeruleus. **G** A representative image of the neonatal medulla (case ID: N9). Intense NLGN4 signals were observed in the hypoglossal nucleus (black arrow) and the dorsal motor nucleus of the vagus (white arrow). Conspicuous NLGN4 signals were also observed in the medial accessory olivary nucleus (black arrowheads). **H** A higher-magnification view of the olivary nucleus in another neonatal brain (case ID: N10). NLGN4 signals were detected at various intensities in neurons of the olivary nucleus, as in pyramidal neurons of the adult cerebral cortex and of the adult hippocampus. Scale bars = 100 µm (**A**, **C**–**E**, **H**), 50 µm (**B**, **F**), 1 mm (**G**)

In the cerebellum, Purkinje cells were scarcely labeled by the antibodies in adult brains (Fig. 6A). In contrast, the Purkinje cells in all neonatal and infantile brains were positive for NLGN4, but the signals were not very intense (Fig. 6B). Granule cells in the cerebellar cortex were negative or scarcely positive for NLGN4 in most adult cases and all neonatal cases. Some larger cells, putative Golgi cells, in the granular layer were occasionally labeled by the antibodies (Fig. 6A). Neurons in the dentate nucleus were also labeled in all adult brains and in most neonatal and infantile brains (not shown). The staining pattern of the dentate nucleus was similar to that of the olive nucleus shown below.

Among the nuclei and other neuronal structures in the pons, neurons in the locus coeruleus were intensely labeled by the antibodies in most of the infantile and adult brains (Fig. 6C–F; Additional file 2: Fig. S2G, H). In addition, neurons in the raphe nucleus of both infantile and adult brains were highly positive for NLGN4 (not shown). Neurons in cranial nerve nuclei of the pons were also positive for NLGN4, but the intensities were not as remarkable as in those described above (not shown).

In the medulla, neurons in the hypoglossal nucleus and the dorsal motor nucleus of the vagus were highly positive for NLGN4 (Fig. 6G). The olivary nucleus showed a unique staining pattern; large neurons were positive for NLGN4 at various intensities, from scarcely detectable to high levels (Fig. 6H). In contrast, neurons in the medial accessory nucleus were consistently positive (Fig. 6G).

### The majority of OXT/AVP-producing neurons in the hypothalamus express NLGN4

**Fig. 7:**
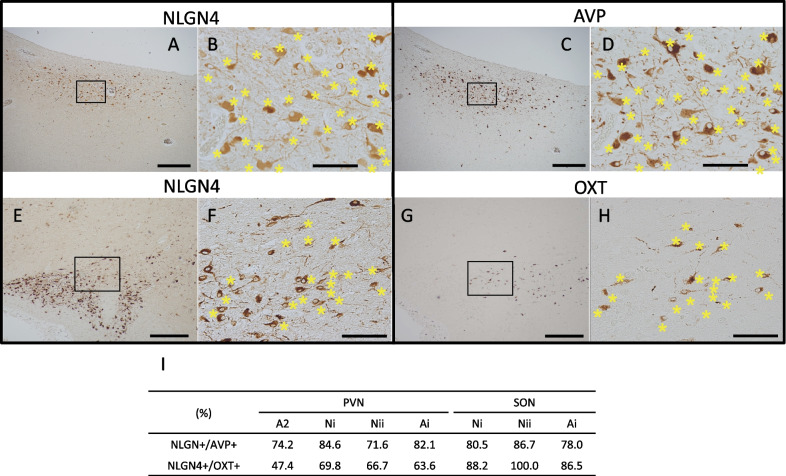
The majority of oxytocin-producing neurons and arginine vasopressin-producing neurons in the human hypothalamus express NLGN4. **A**–**D** Representative paired images of NLGN4 distribution (**A**, **B**) and arginine vasopressin (AVP) distribution (**C**, **D**) in the adult PVN (case ID: A2). Mirror sections were prepared and immunostained with either anti-NL4-p1 antibodies or anti-AVP antibodies. **A**, **C** Low-magnification views. **B**, **D** Magnified views of the boxed areas in **A** and **C**, respectively. Asterisks indicate cells regarded as identical in **B** and **D**. **E**–**H** Representative paired images of NLGN4 distribution (**E**, **F**) and oxytocin (OXT) distribution (**G**, **H**) in the neonatal SON (case ID: Ni). **E**, **G** Low-magnification views. **F**, **H** Magnified views of the boxed areas in **E** and **G**, respectively. Asterisks indicate cells regarded as identical in **F** and **H**. Scale bars = 500 µm (**A**, **C**, **E**, **G**), 100 µm (**B**, **D**, **F**, **H**). **I** Ratios of NLGN4-positive neurons among AVP-positive neurons (NLGN4 + /AVP +) and NLGN4-positive neurons among OXT-positive neurons (NLGN4 + /OXT +) in the adult and neonatal PVN and SON. The values are shown as percentages. Details of cases Ni, Nii, and Ai are described in the text

We were interested in intensive NLGN4 signals in the PVN and SON, since these nuclei are the centers producing social neuropeptides, oxytocin (OXT) and arginine vasopressin (AVP) and impaired OXT/AVP system can be involved in the pathophysiology of ASD. We therefore examined whether NLGN4-positive cells in the PVN and SON produce these neuropeptides using mirror sections. Because of the limitation of archival autopsy samples, we could perform this additional analysis only on the PVN of adult case A2. As shown in Fig. 7A–D, most of AVP-positive cells appeared NLGN4-positive. In contrast, less OXT-positive cells expressed NLGN4 in this case (not shown). Then we quantified the percentages of NLGN4-positive cells among AVP-positive cells and OXT-positive cells. Quantification analyses revealed that approximately three-quarters of AVP-positive neurons expressed NLGN4, while less than half of OXT-positive neurons expressed NLGN4 in case A2 (Fig. 7I). To obtain further information about the proportion of NLGN4-expressing cells among AVP/OXT-producing neurons, we additionally examined PVNs and SONs in cases that had been excluded from the study owing to the presence of neurodevelopmental or neurological disorders. Three PVNs and SONs without histological abnormalities were available from two neonatal cases, Ni (diagnosed as 4p-syndrome (Fig. 7E–H)) and Nii (unspecified multiple anomalies), and one adult case, Ai (diagnosed as Parkinson’s disease). Much less OXT-producing neurons (Fig. 7G) compared with NLGN4-positive neurons (Fig. 7E) were considered due to the fact that the great majority (90% or more than 95%) of the neurosecretory neurons were AVP-producing neurons, while OXT-producing neurons were very minor population in SON [27, 28]. Yet most of the scattered OXT-producing neurons appeared NLGN4-positive (Fig. 7F, H). In these three cases, 71.6–84.6% of AVP-positive neurons in PVN and 78.0–86.7% of those in SON were NLGN4 positive and 63.6–69.8% of OXT-positive neurons in PVN and 86.5–100% of those in SON were NLGN4 positive (Fig. 7I). Thus, we concluded that the majority of OXT-producing neurons as well as AVP-producing neurons in both the PVN and SON were considered to express NLGN4 (Fig. 7I).

## Discussion

We established two kinds of antibodies against unique amino acid residues of NLGN4 and confirmed that they were highly specific for NLGN4 among the extremely conserved NLGN protein family. Utilizing these antibodies, we examined the distribution of NLGN4-positive cells in the human brain. This study revealed a couple of unique and intriguing features of NLGN4, which may be relevant to the neuronal functions impaired in ASD.

Among various subtypes of neurons that express NLGN4, neurons functioning in intellectual abilities, i.e., the cerebral cortical and hippocampal neurons; in social abilities, i.e., neurons in the PVN and SON; in sensory processing, i.e., the thalamic neurons; and in the regulation of wakefulness and consciousness, i.e., neurons in the locus coeruleus and the raphe nucleus, were found to express NLGN4 constantly at high levels (Table 2). In contrast, neurons functioning in motor processing, i.e., neurons in the putamen, globus pallidus, substantia nigra, red nucleus, and cerebellar cortex, expressed NLGN4 not at all or very weakly, especially in the mature brain. Such preferential distribution firmly supports that NLGN4 is involved, especially in intellectual, social, sensory, and consciousness functions in the brain, while it is less involved in motor function. This seemingly explains why the disruption of the *NLGN4X* gene results in autism accompanying intellectual disability [2, 3]. It is intriguing that neurons expressing NLGN4 intensely were identified exceptionally in the motor nuclei of the cranial nerves, i.e., the oculomotor nucleus and the hypoglossal nucleus, since the former is important for normal visual attention and the latter is involved in speaking ability, both of which are affected in autism.

Intensive expression of NLGN4 in OXT/AVP-producing neurons is especially noteworthy, since it can be tightly relevant to impaired social function, which is one of the major symptoms of ASD. OXT has emerged as a neuromodulator of diverse social behaviors, and extensive studies have been conducted on its possible pathophysiological involvement and therapeutic effects in ASD, yet many controversial issues have remained [29–32]. At least its therapeutic effects in ASD were questioned, since the most recent, large-scale, placebo-controlled trial of intranasal oxytocin therapy in ASD patients showed no significant effects on social or cognitive function of them [33]. AVP and OXT confer mostly opposite effects on anxiety and depression-related behavior; however, they have effects in the same direction on social behavior [34]. Therefore, AVP is considered another potential biomarker as well as a therapeutic reagent for ASD; promising data in support of this hypothesis have been accumulated recently [35–37]. Our quantitative examination revealed that the majority of AVP-producing cells and OXT-producing cells in both the PVN and SON expressed NLGN4, with the exception of OXT-producing cells in the PVN of case A2. We could not completely rule out the possibility that the expression of AVP, OXT, and/or NLGN4 was affected somehow by the disease conditions in additional cases; however, significant changes seemed improbable. Thus, it is suggested that NLGN4 functions in the majority of social neuropeptide-producing neurons.

Intriguingly, our data demonstrated that the subcellular distribution of NLGN4 in neurons appeared as a cytoplasmic pattern but not as a surface membrane protein pattern. This is consistent with previous studies that reported cytoplasmic localization of other NLGNs [24–26], and the widespread cytoplasmic distribution seems to be a common feature of NLGNs despite their primary emergence as synaptic cell-adhesion molecules interacting with their binding partner NRXNs [11, 12]. We tried to obtain further information about the subcellular distribution of NLGN4 by immunofluorescent multiple staining, but archival brain samples did not provide sufficient resolution to distinguish subcellular organelles. Recently, Nguyen et al. reported that autism-associated variants of NLGN4X have deficits in trafficking to the cell surface, phenocopying NLGN4Y [15]. They demonstrated that NLGN4Y did not efficiently traffic to the cell surface, which hindered its ability to induce synapses. They also suggested that NLGN4Y was distinct from NLGN4X regardless of their extreme structural similarities and that it could act as a negative regulator of other NLGNs heterodimerizing with them. Since the antibodies we generated and applied in this study were designed to target peptide sequences that were almost identical in NLGN4X and NLGN4Y, they might react with both NLGN4X and NLGN4Y. Therefore, it is possible that the cytoplasmic NLGN4 signals were derived from NLGN4Y that failed to traffic to the cell surface in male cases; however, this should not occur in female cases. We observed a widespread cytoplasmic distribution of NLGN4 signals equally in male and female cases, as presented in the results, and insist that a considerable fraction of NLGN4X localizes in the cytoplasm, independent of NLGN4Y expression. Intriguingly, OXT/AVP-producing neurons secrete OXT/AVP from their axon terminals into plasma and, in independently regulated manners, also release OXT/AVP from dendrites locally in the brain, which may induce many behavioral effects [38–40]. It is tempting to speculate that the transmembrane protein NLGN4, which is distributed throughout the cytoplasm of OXT/AVP-producing neurons, might localize to small vesicles involved in dendritic secretion of neuropeptides. Further studies on the nonsynaptic functions of NLGN4 are necessary and important to understand the entire roles of this transmembrane protein in the brain.

## Supplementary Information

**Additional file 1: Figure S1. **Specificity of anti-NL4-m1 antibodies and generation of *NLGN4X* knockout human iPSC clones (hiPSC NL4-KO). (A) Anti-NL4-m1 antibodies specifically react with NLGN4 but not with the other NLGNs in Western blotting. Lysates of HEK293 cells expressing FLAG-tagged NLGN1 (FLAG-NL1), NLGN2 (FLAG-NL2), NLGN3 (FLAG-NL3), or NLGN4 (FLAG-NL4) were electrophoresed and immunoblotted with anti-FLAG and anti-NL4-m1 antibodies. Anti-NL4-m1 antibodies reacted with FLAG-NL4 and did not cross-react with FLAG-NL1, 2, and 3. (B) Schematic structures of the partial human *NLGN4X* gene (upper schematic diagram) and an expected edited gene in the hiPSC NL4-KO clone (KO: lower schematic diagram). The hiPSC NL4-KO clone was generated by engineering the *NLGN4X* gene of human iPSC clone 610B1 using the CRISPR/Cas9 system. Target sites of two guide RNAs (gRNA1 and gRNA2) are indicated by white arrows. Positions of primer sets (pEx2f and r, pEx6f and r) flanking the target sites to confirm expected gene editing and sizes of respective PCR amplicons are also indicated in the schema. (C) Agarose gel electrophoresis images of PCR products from human iPSC clone 610B1 (hiPSC WT) genomic DNA or hiPSC NL4-KO genomic DNA. Expected PCR products were amplified from hiPSC WT genomic DNA with primer sets pEx2f/pEx2r and pEx6f/pEx6r but not from hiPSC NL4-KO genomic DNA. In contrast, PCR products were amplified only from hiPSC NL4-KO genomic DNA with the primer set pEx2f/pEx6r. These data are compatible with the finding that the hiPSC NL4-KO clone has the *NLGN4X* gene with targeted deletion spanning from the middle of exon 2 to the middle of exon 6. As an internal control, the X-linked gene PUDP was used.

**Additional file 2: Figure S2**. Additional images stained with anti-NL4-p1 and anti-NL4-m1. (A, B) Low magnification views of a neonatal paraventricular nucleus (case ID: N1) stained with either anti-NL4-p1 (A) or anti-NL4-m1 (B). Similar staining patterns are confirmed, yielding a higher signal-to noise ratio by anti-NL4-p1. (C, D) Low magnification views of a neonatal supraoptic nucleus (case ID: N1) stained with either anti-NL4-p1 (C) or anti-NL4-m1 (D). Similar staining patterns were confirmed. (E, F) Low magnification views of adult oculomotor nuclei (case ID: A7) stained with either anti-NL4-p1 (E) or anti-NL4-m1 (F). Intense signals are observed in neurons by anti-NL4-p1, while weak signals are identified in neurons by anti-NL4-m1. (G, H) Low magnification views of an adult locus coeruleus (case ID: A4) stained with either anti-NL4-p1 (G) or anti-NL4-m1 (H). Similar staining patterns are confirmed, yielding a higher signal-to noise ratio by anti-NL4-p1. Scale bars = 250 µm (A-H).

## Data Availability

Materials including antibodies and *NLGN4* KO iPSC clones are available on request for noncommercial use.

## Abbreviations

AVP: Arginine vasopressin
iPSC: Induced pluripotent stem cell
NLGN: Neuroligin
NRXN: Neurexin
OXT: Oxytocin
PVN: Paraventricular nucleus
SON: Supraoptic nucleus
