# Supplementary figures and images for: The distribution of neuroligin4, an autism-related postsynaptic molecule, in the human brain
Source: Mol Brain. 2023 Feb 6;16:20. doi: 10.1186/s13041-023-00999-y (PMC9903511; doi:10.1186/s13041-023-00999-y)

## Slide 1
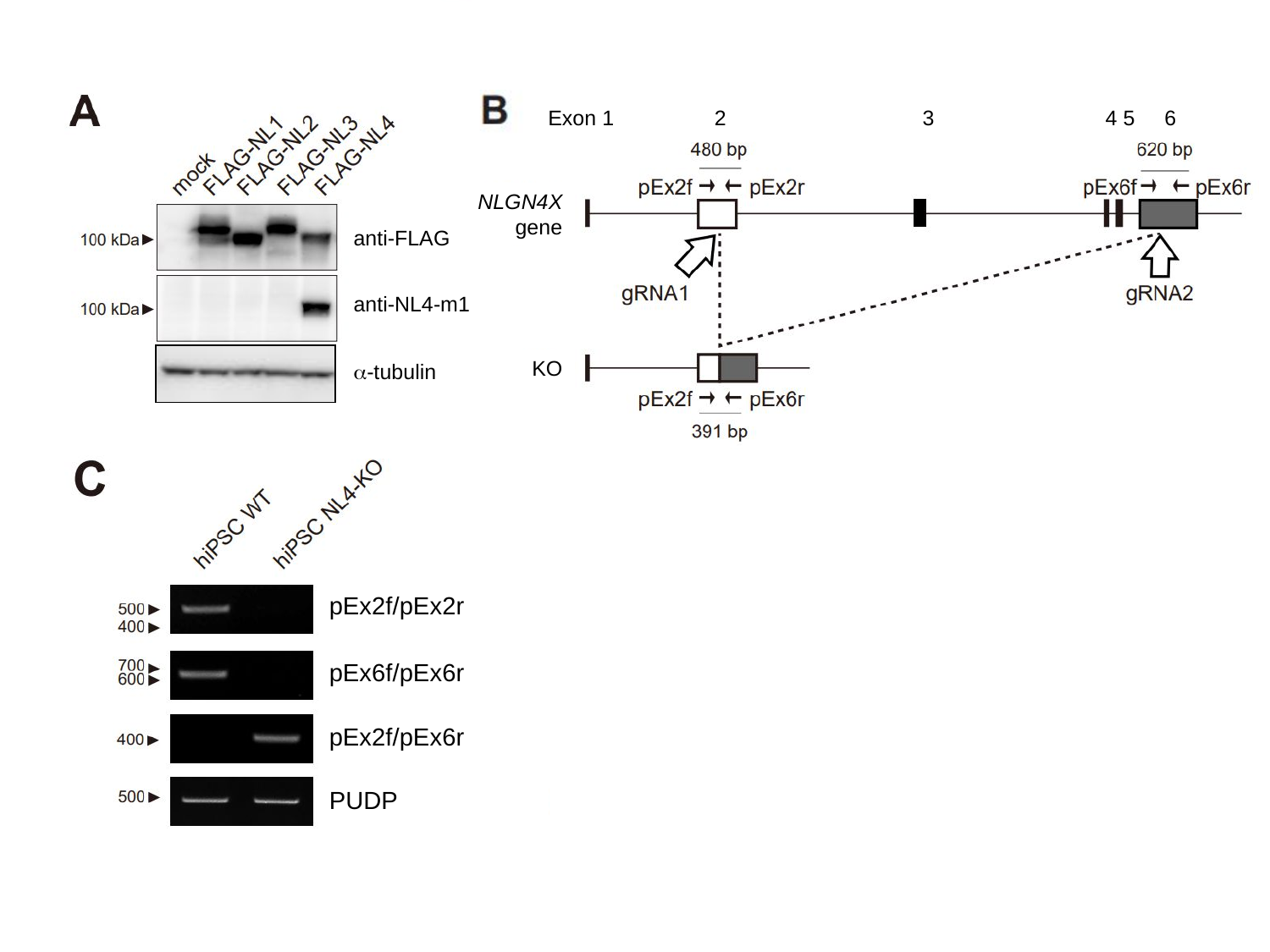

Exon 1 2 　 3 4 5 6
NLGN4X
gene
KO
a-tubulin
anti-FLAG
anti-NL4-m1
pEx2f/pEx2r
pEx6f/pEx6r
pEx2f/pEx6r
PUDP

Supplement: Supplementary file 1 — Additional file 1: Figure S1. Specificity of anti-NL4-m1 antibodies and generation of NLGN4X knockout human iPSC clones (hiPSC NL4-KO). (A) Anti-NL4-m1 antibodies specifically react with NLGN4 but not with the other NLGNs in Western blotting. Lysates of HEK293 cells expressing FLAG-tagged NLGN1 (FLAG-NL1), NLGN2 (FLAG-NL2), NLGN3 (FLAG-NL3), or NLGN4 (FLAG-NL4) were electrophoresed and immunoblotted with anti-FLAG and anti-NL4-m1 antibodies. Anti-NL4-m1 antibodies reacted with FLAG-NL4 and did not cross-react with FLAG-NL1, 2, and 3. (B) Schematic structures of the partial human NLGN4X gene (upper schematic diagram) and an expected edited gene in the hiPSC NL4-KO clone (KO: lower schematic diagram). The hiPSC NL4-KO clone was generated by engineering the NLGN4X gene of human iPSC clone 610B1 using the CRISPR/Cas9 system. Target sites of two guide RNAs (gRNA1 and gRNA2) are indicated by white arrows. Positions of primer sets (pEx2f and r, pEx6f and r) flanking the target sites to confirm expected gene editing and sizes of respective PCR amplicons are also indicated in the schema. (C) Agarose gel electrophoresis images of PCR products from human iPSC clone 610B1 (hiPSC WT) genomic DNA or hiPSC NL4-KO genomic DNA. Expected PCR products were amplified from hiPSC WT genomic DNA with primer sets pEx2f/pEx2r and pEx6f/pEx6r but not from hiPSC NL4-KO genomic DNA. In contrast, PCR products were amplified only from hiPSC NL4-KO genomic DNA with the primer set pEx2f/pEx6r. These data are compatible with the finding that the hiPSC NL4-KO clone has the NLGN4X gene with targeted deletion spanning from the middle of exon 2 to the middle of exon 6. As an internal control, the X-linked gene PUDP was used. [file 13041_2023_999_MOESM1_ESM.pptx]

## Slide 1
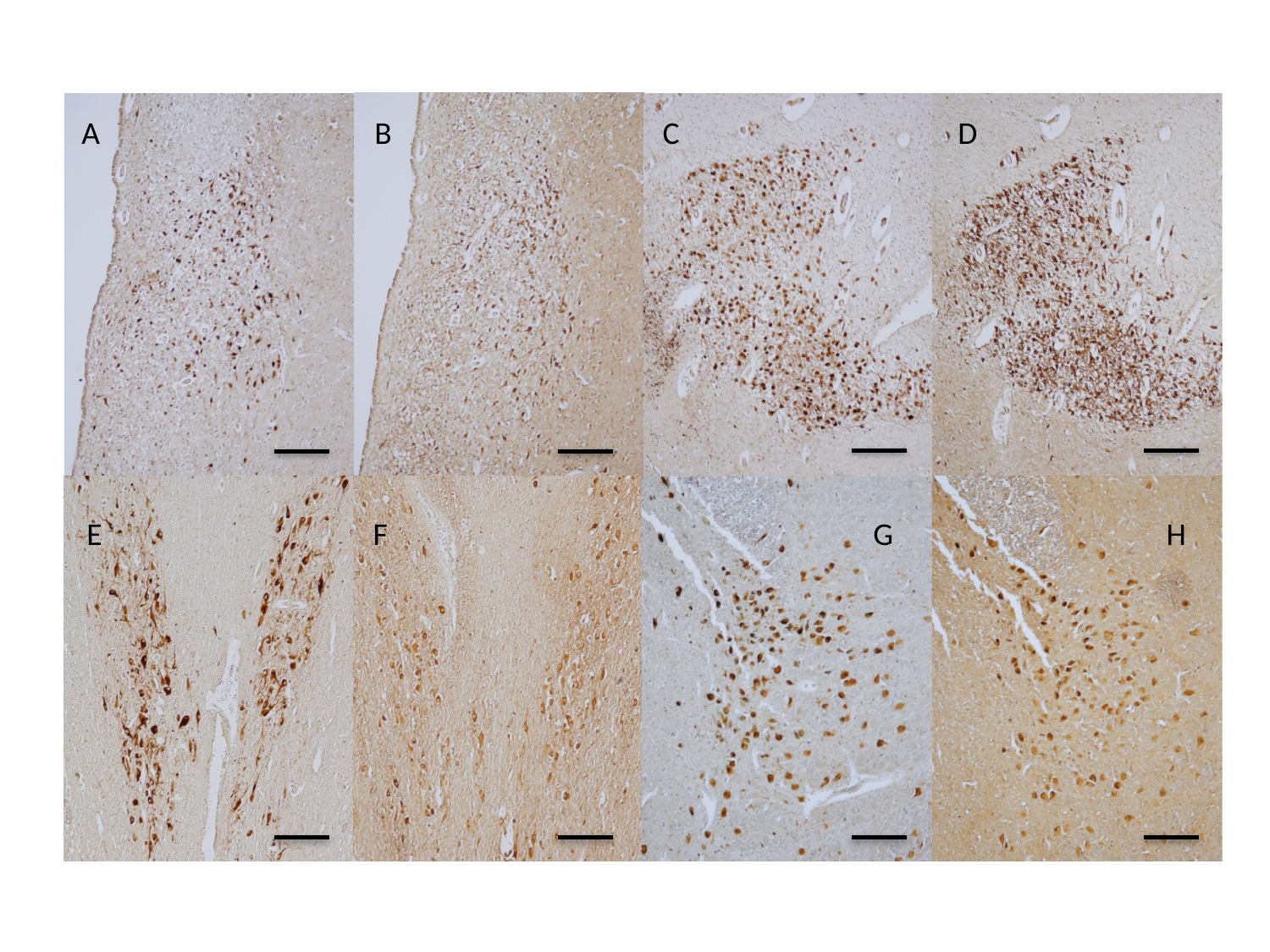

A
B
D
C
E
F
G
H

Supplement: Supplementary file 2 — Additional file 2: Figure S2. Additional images stained with anti-NL4-p1 and anti-NL4-m1. (A, B) Low magnification views of a neonatal paraventricular nucleus (case ID: N1) stained with either anti-NL4-p1 (A) or anti-NL4-m1 (B). Similar staining patterns are confirmed, yielding a higher signal-to noise ratio by anti-NL4-p1. (C, D) Low magnification views of a neonatal supraoptic nucleus (case ID: N1) stained with either anti-NL4-p1 (C) or anti-NL4-m1 (D). Similar staining patterns were confirmed. (E, F) Low magnification views of adult oculomotor nuclei (case ID: A7) stained with either anti-NL4-p1 (E) or anti-NL4-m1 (F). Intense signals are observed in neurons by anti-NL4-p1, while weak signals are identified in neurons by anti-NL4-m1. (G, H) Low magnification views of an adult locus coeruleus (case ID: A4) stained with either anti-NL4-p1 (G) or anti-NL4-m1 (H). Similar staining patterns are confirmed, yielding a higher signal-to noise ratio by anti-NL4-p1. Scale bars = 250 µm (A-H). [file 13041_2023_999_MOESM2_ESM.pptx]
